# Expression of Concern: ACKR4 restrains antitumor immunity by regulating CCL21

**DOI:** 10.1084/jem.2019063411202023e

**Published:** 2023-11-28

Based on external and internal investigations conducted by QIMR Berghofer Medical Research Institute in response to concerns around data fabrication and falsification by Professor Mark Smyth, *JEM* is issuing this Expression of Concern regarding Figures 4 A, 4 I, 5 A, 5 B, S2 D, and S2 F from Whyte et al. (2020) *J. Exp. Med.* 217 (6): e20190634.

Vol. 217, No. 6 | https://doi.org/10.1084/jem.20190634 | April 14, 2020
